# Evaluating the long-term cost-effectiveness of fixed-ratio combination insulin degludec/liraglutide (IDegLira) versus other treatment regimens in the chinese type 2 diabetes patients

**DOI:** 10.1186/s13098-023-01141-7

**Published:** 2023-08-19

**Authors:** Ran Wei, Weihao Wang, Xiusheng Huang, Jingtao Qiao, Jinghe Huang, Chang Xing, Qi Pan, Lixin Guo

**Affiliations:** 1 Department of Endocrinology, Beijing Hospital, National Center of Gerontology, Institute of Geriatric Medicine, Chinese Academy of Medical Sciences, Beijing, PR China; 2 Peking University Fifth School of Clinical Medicine, Beijing, China; 3 Novo Nordisk (China) Pharmaceuticals Co., Ltd, Beijing, China; 4 Department of Orthopedics, The First Affiliated Hospital of Zhengzhou University, Zhengzhou City, Henan Province PR China

**Keywords:** Cost-effectiveness, IDegLira, China, Type 2 diabetes

## Abstract

**Background and aims:**

To assess the cost-effectiveness of utilizing IDegLira in comparison to other treatment regimens ( liraglutide and degludec) in managing type 2 diabetes, taking into account the Chinese healthcare system’s perspective.

**Methods:**

The clinical data were obtained from the randomized controlled trials (RCTs) of the DUAL I and DUAL II evidence studies that took place in China. To estimate the lifetime quality-adjusted life-years (QALYs) and direct medical costs of patients receiving different treatment strategies from a long-term perspective, the IQVIA CORE Diabetes Model version 9.0 (IQVIA, Basel, Switzerland) was utilized. The costs were evaluated from the perspective of the China National Health System. Future costs and clinical benefits were discounted annually at 5%, and sensitivity analyses were conducted.

**Results:**

IDegLira was projected to reduce the incidence of diabetes-related complications and improve quality-adjusted life expectancy (QALE) versus liraglutide and degludec. A survival benefit was observed with IDegLira over Liraglutide (0.073 years). Lifetime costs were lower by Chinese yuan (CNY) 27,945 on IDegLira than on Liraglutide therapy. A similar survival benefit was observed with IDegLira over degludec (0.068 years). Lifetime costs were lower by CNY 1196 on IDegLira than on degludec therapy. Therefore, IDegLira was found to be cost-effective versus liraglutide and degludec with incremental cost-effectiveness ratios of Dominant per QALY gained, respectively, under the threshold of three times the gross domestic product (GDP) per capita in China.

**Conclusion:**

IDegLira is a cost-effective hypoglycemic treatment option that delivers positive clinical outcomes while also reducing costs for Chinese patients living with type 2 diabetes.

**Supplementary Information:**

The online version contains supplementary material available at 10.1186/s13098-023-01141-7.

## Introduction

Type 2 diabetes mellitus (T2DM) is a chronic health condition. As per the 9th edition of the International Diabetes Federation’s global diabetes map, approximately 116 million individuals in China had diabetes in 2019, and this number is anticipated to rise to 147 million by 2045, with the majority of cases being T2DM [1]. Costs, ranged from $673 billion to $1,197 billion in 2015, and are expected to exceed $802 billion to $1,452 billion by 2040 [2]. Due to the fast-paced aging of the Chinese population, diabetes has become a significant contributor to the overall burden on the healthcare system. For Chinese healthcare providers, the economic strain associated with diabetes has become an ongoing challenge [3].

IDegLira (Xultophy®) is a once-daily injected combination of insulin degludec and liraglutide in a fixed proportion. Insulin degludec is a basal insulin therapy with a half-life of over 24 h, while liraglutide is a glucagon-like peptide-1 (GLP-1) receptor agonist. This combination is an option for type 2 diabetes patients who are unable to achieve their glycemic goals (HbA1c > 7.5%) with basal insulin alone. The combination benefits from the complementary mechanisms of action of both drugs, with GLP-1 receptor agonists reducing some adverse effects associated with basal insulin therapy, particularly hypoglycemia and weight gain [4]. IDegLira’s Global DUAL Clinical Trial Program I-IX [5–15] has demonstrated effective glycemic control in patients with type 2 diabetes, leading to approval for management of the condition in Europe and the United States. Novo Nordisk has developed and produced IDegLira, which has recently been approved by the China National Medical Products Administration, specifically for patients with poor blood sugar control with a baseline HbA1c level of 7.5% or higher on basal insulin and metformin [16].

Compared with its components given alone, IDegLira significantly reduces HbA1c and weight loss, with the lower incidence of diabetes-related complications in the long term [5]. For healthcare payers, this study aims to provide essential information by evaluating the long-term impact on treatment efficacy and quality of life. The analysis focuses on assessing the cost-effectiveness of IDegLira compared to other treatment regimens (liraglutide, degludec) for poorly controlled patients with type 2 diabetes who are currently receiving basic insulin therapy, from the perspective of a Chinese healthcare payer.

## Materials and methods

### Cost-effectiveness analysis

#### Model overview

Taking place between May 2017 and December 2018, DUALI China included 720 patients. It followed up 26-week. The mean age is 54.7 (10.3) year, while mean Hba1c is 8.23% (0.82). Taking place May 2017 to July 2019,DUALII China included 453 patients.It followed up 26-week.The mean age is 54.7 (9.9) year, while mean Hba1c is 8.94% (1.19). DUALI was used in base case, while DUAL II was used for scenario analysis. Long-term projections of costs and clinical outcomes based on data from the DUALI [17] and DUALII [16] China clinical trial study were made using the IQVIA CORE Diabetes Model Version 9.0 (IQVIA, Basel, Switzerland), a previously published and validated model of type 2 diabetes [18–20]. The non-product-specific diabetes policy analysis tool is capable of conducting real-time simulations that incorporate different treatment regimens. It projected life expectancy, quality-adjusted life expectancy, complication rates, time to onset of complications, and direct costs through 1000 iterations of individual cohorts, each containing 1000 simulated patients. For the base case and one-way sensitivity analysis, they ran first-order Monte Carlo simulations (also known as random walk or microsimulations), which included probabilistic sensitivity analysis, with separate presentations of sampling patients’ baseline characteristics, treatment effects, probabilities, costs, and utilities from distributions in the model. They evaluated cost-effectiveness by computing incremental cost-effectiveness ratios (ICERs) where appropriate. Probabilistic sensitivity analysis generated cost-effectiveness scatterplots and acceptability curves to assess base case outcome uncertainty. The base case analyses utilized a 40-year time horizon. They discounted future costs and clinical benefits at a rate of 5% per annum, in accordance with published guidance for China [21].

#### Model inputs

##### Baseline cohort characteristics

We carefully selected a group of simulated patients based on the initial levels of Chinese individuals with type 2 diabetes mellitus who participated in the DUALI China clinical trial [17]. Additional missing information stems from the references [22]. Comprehensive baseline cohort characteristics are outlined in Appendix S1 and S2.

##### Treatment outcomes

The treatment effects assessed in our analysis consisted of the baseline differences in HbA1c and body mass index as well as rates of hypoglycaemia events. The treatment effects for the IDegLira group compared to the liraglutide and degludec group were obtained from the DUALI China clinical trial [17]. We did not take into account the impact of the treatment switching pattern. Table 1 presents the input variables for the treatment effect in each group.

**Table 1:** Treatment effects applied in the analysis

	HbA1c change from baseline (%)	BMI change from baseline (%)	Non-severe hypoglycaemia (events per 100 patient-year)	Source
IDegLira	-1.66	0.04	24.00	DUAL I China
liraglutide	-1.04	-0.86	4	DUAL I China
degludec	-1.13	0.43	17	DUAL I China

Patients who were prescribed IDegLira, liraglutide, or degludec continued on this treatment until their HbA1c levels rose above 7.5%. At that point, they were switched to basal-bolus therapy with IGlar OD and 3 doses of IAsp [23].

##### Costs

The model considered direct medical costs, which encompassed drug acquisition expenses, treatment costs for diabetes-related complications, and routine patient management expenses. All costs were denominated in CNY as of the year 2023. The China Hospital Pharmaceutical Audit database was the source of drug acquisition expenses. The daily costs of IDegLira, degludec, and liraglutide were 17.7, 8.3, and 31.53 CNY, respectively.

The expenses associated with complications related to diabetes during the year of occurrence, and subsequent annual follow-up costs (each year of the simulation after the event) have been outlined in Appendix S1 and S2. These were primarily determined by estimating the direct medical expenditures for diabetes-related complications, based on the sampling claims data gathered by the China Health Insurance Research Association (CHIRA) [24]. The cost information not included in the CHIRA database was collected from other Chinese literature [25].

##### Utilities and disutilities

Health utility values and disutility associated with T2DM and its complications were obtained from literature sources [26–29].

##### Base-case analysis

Cost-effectiveness was evaluated through the calculation of ICERs where appropriate. Probabilistic sensitivity analysis was carried out to generate cost-effectiveness scatter plots and acceptability curves, which were used to assess uncertainty around the base case outcomes. For the base-case analyses, a time horizon of 40 years was utilized, with future costs and clinical benefits being discounted at an annual rate of 5%, as per published guidance for China.

According to Dr Men’s research [30], the willingness to pay (WTP) for a QALY in China is three times its GDP per capita. Thus, the WTP was calculated as 257,094 CNY per QALY in 2022, based on triple the country’s GDP.

##### Sensitivity analysis

One-way and probabilistic sensitivity analyses were conducted for each of the two subgroups to identify the key variables that influenced costs and clinical outcomes. The one-way sensitivity analysis involved varying the treatment switch at either 3 or 5 years, the HbA1c time at 8.5% or 7%, the horizon at either 20 or 30 years, discount rates ranging from 0 to 8%, and changes in complication costs and utilities by 25% or 5%.

##### Scenario analysis

Scenario analysis involves estimating the anticipated outcome of a particular change in key factors [31]. Scenario analysis involves estimating the anticipated outcome of a particular change in key factors [16]. Detailed baseline cohort characteristics are listed in Appendix S4.

## Results

### IDegLira versus liraglutide

**Fig. 1:**
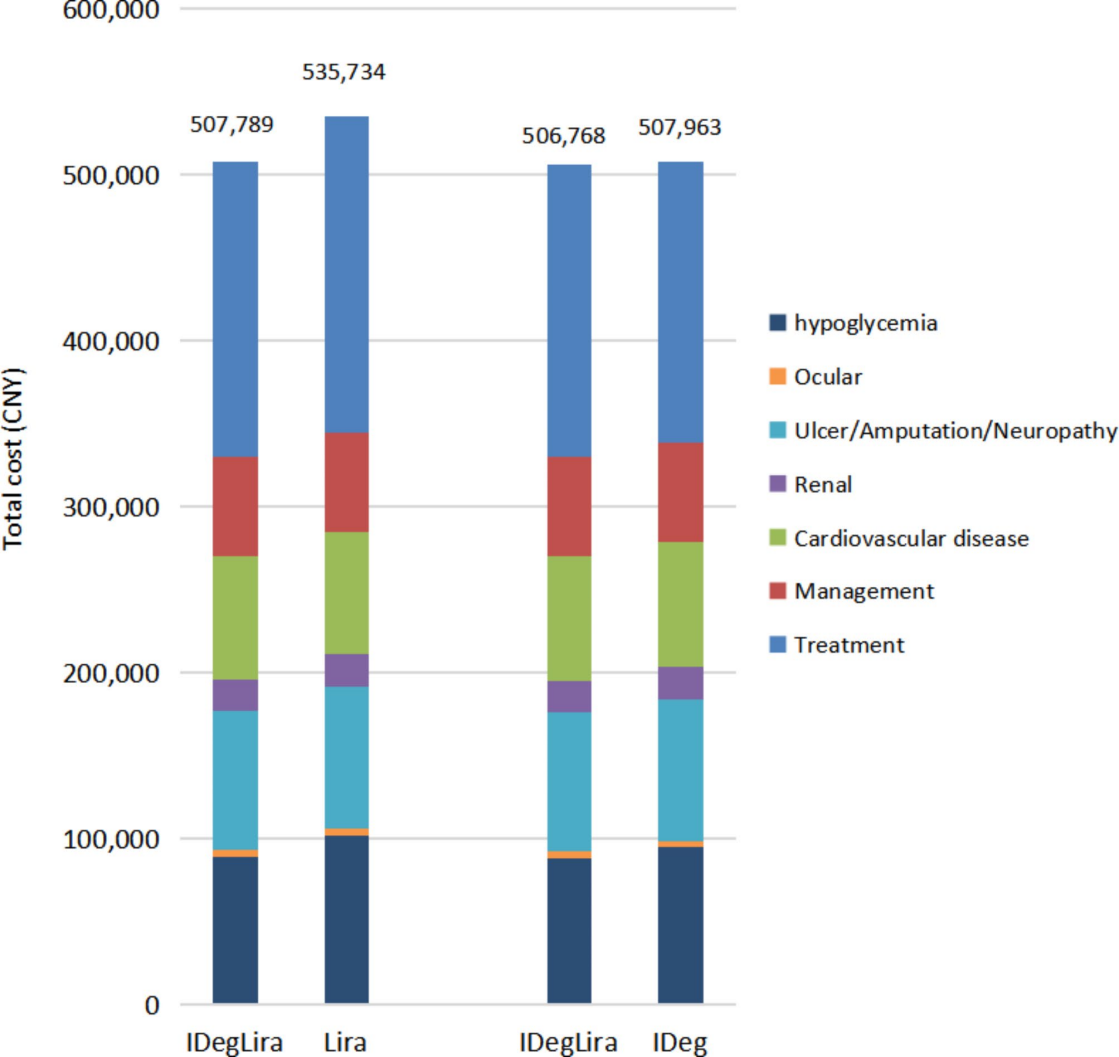
Breakdown of direct costs by cost category. Costs were categorized as treatment costs (costs associated with diabetes therapy), management costs (associated with routine care) and complication costs (associated with cardiovascular, renal, diabetic foot or neuropathy, or ocular complications)

This resulted in an increase of 0.073 QALYs for IDegLira compared to Liraglutide (Tables 2 and 3). The total cost of lifelong treatment with IDegLira was lower by CNY 27,945 than Liraglutide therapy (CNY 507,788.938 vs. CNY 535,734.375), as the higher pharmacy costs in the IDegLira group were partly balanced out by the reduced costs of diabetes-related complications (Fig. 1). IDegLira showed a Dominant ICER per QALY gained versus Liraglutide.

**Table 2:** Main results of the base-case cost-effectiveness analysis. Abbreviations: CNY, Chinese yuan

	IDegLira (mean [SD])	Liraglutide (mean [SD])	IDegLira (mean [SD])	degludec (mean [SD])
Life expectancy (years)	14.007 (0.114)	13.975 (0.111)	13.952 (0.109)	13.909 (0.115)
Quality-Adjusted Life expectancy (years)	12.081 (0.098)	12.007 (0.096)	12.031 (0.093)	11.963 (0.100)
Direct costs(CNY)	507788.938 (6972.7)	535734.375 (7272.03)	506767.563 (6,950.79)	507,963.13 (6,726.01)

**Table 3:** Summary results of the base-case cost-effectiveness analysis. Abbreviations: QALY, quality-adjusted life-year; ICER, incremental cost-effectiveness ratio

	IDegLira vs. Liraglutide	IDegLira vs. degludec
ΔQALY	0.073	0.068
ΔCOST	-27,945	-1196
ICER	Dominant	Dominant

### IDegLira versus degludec

This resulted in an increase of 0.068 QALYs with IDegLira compared to degludec (Tables 2 and 3). Lifetime expenses on IDegLira were lower by CNY 1196 when compared to degludec therapy (CNY 506767.563 vs. 507,963.13) with the higher pharmacy costs in the IDegLira group being partially balanced out by the reduced expenses on diabetes-related complications (Fig. 1). IDegLira showed an ICER of Dominant per QALY gained, when compared to degludec.

### Sensitivity analyses

**Fig. 2:**
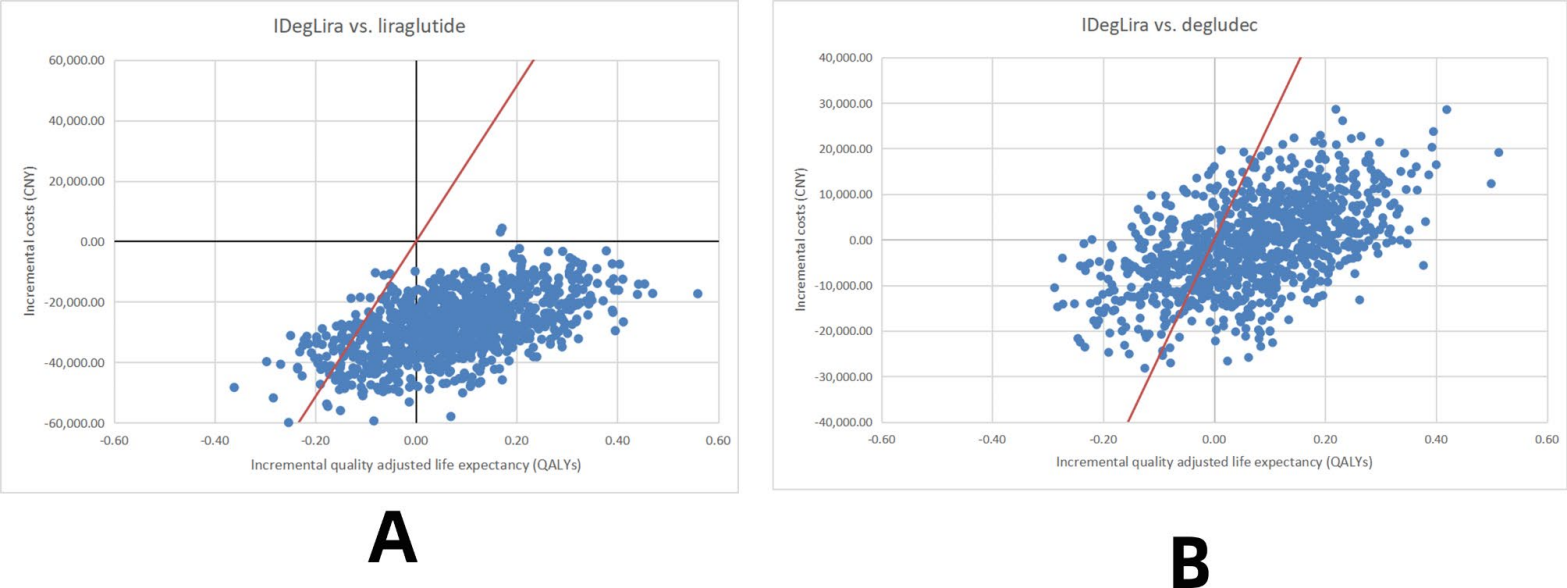
Probabilistic sensitivity analysis cost-effectiveness scatterplots for the two subgroups based on DUAL I China (**a**) compared with liraglutide, (**b**) compared with degludec. The red line represents a WTP of CNY 257,094 per QALY gained. QALY, quality-adjusted life-year; CNY, Chinese yuan; WTP, willingness to pay

As shown in Fig. 2, the results of the probabilistic sensitivity analysis indicate that with a WTP of CNY 257,094 per QALY gained, IDegLira holds its position as a more cost-effective option as compared to liraglutide and degludec.

**Fig. 3:**
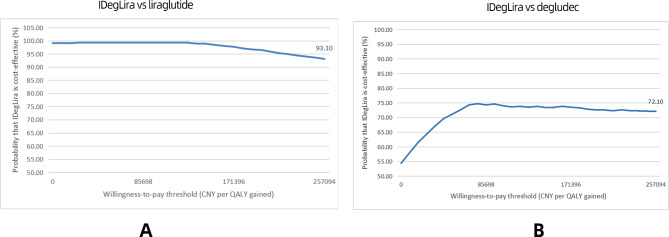
The acceptability of IDegLira based on QALE based on DUAL I China (**a**) compared with liraglutide, (**b**) compared with degludec. QALE ,quality-adjusted life expectancy

As shown in Fig. 3a, the acceptability of IDegLira based on QALE was observed to be more than 80% when compared with liraglutide. Furthermore, in Fig. 3b, when compared with degludec, the acceptability of IDegLira based on QALE was also observed to be greater than 50%.

### Scenario analysis

**Fig. 4:**
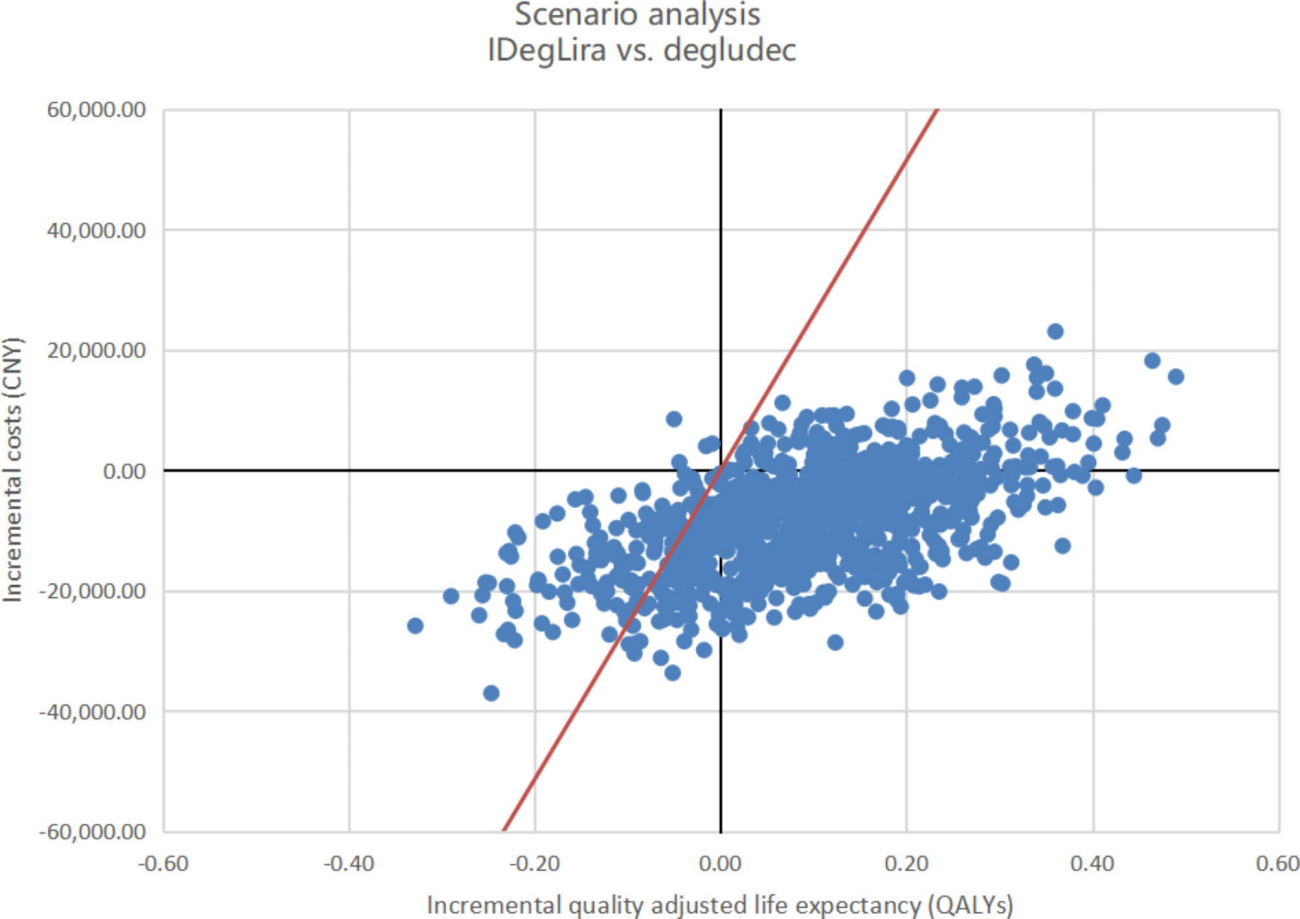
Probabilistic sensitivity analysis cost-effectiveness scatterplots for IDegLira compared with the degludec based on DUAL II China. The red line represents a WTP of CNY 257,094 per QALY gained. QALY, quality-adjusted life-year; CNY, Chinese yuan; WTP, willingness to pay

**Fig. 5:**
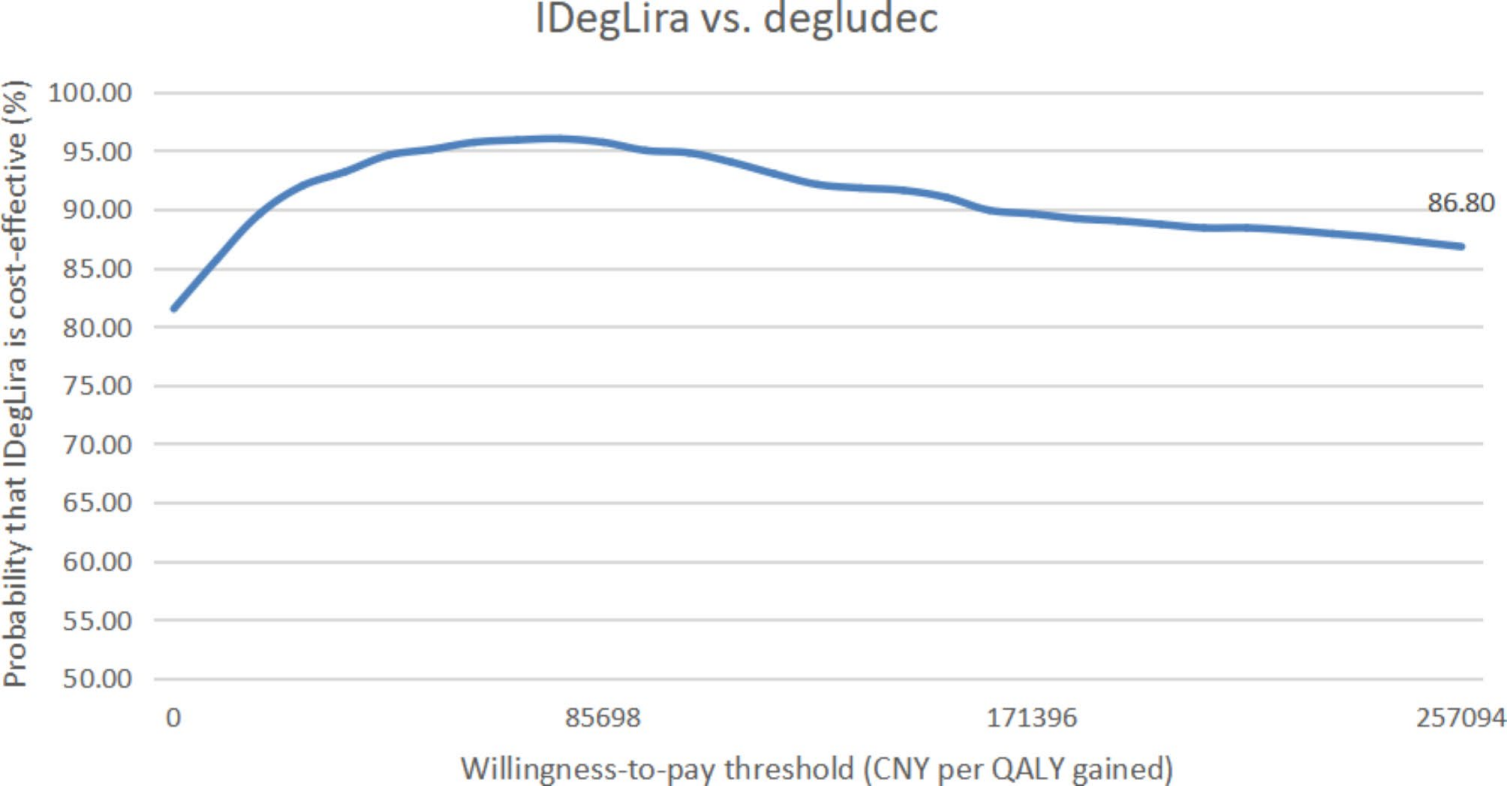
The acceptability of IDegLira based on QALE compared with degludec based on DUAL II China. QALE ,quality-adjusted life expectancy

As illustrated in Fig. 4, a probabilistic sensitivity analysis revealed that IDegLira remained cost-effective compared to degludec, assuming a WTP of CNY 257,094 per QALY gained. Figure 5 shows that the acceptability of IDegLira based on QALE was over 80% when compared to degludec.

## Discussion

As a populous country in the world, China has an enormous number of diabetics, which is constantly surging [32]. Diabetes constitutes a significant aspect of the country’s healthcare burden [33]. The economic burden associated with diabetes continues to increase significantly, posing a persistent challenge for healthcare payers in China. Chinese doctors and healthcare payers must prioritize clinical benefits while also taking cost considerations into account when selecting drugs. IDegLira, a new medication on the Chinese market, was launched in 2022 and was added to China’s medical insurance payment list in February 2023. As far as we know, this is the first long-term cost-effectiveness analysis conducted on IDegLira for diabetic patients in China.

It is vital to note that this model is not the first time we conducted a pharmacoeconomic analysis of IDegLira in China. In fact, back in February of 2022, we attempted a cost-effectiveness analysis (CEA) on IDegLira when it was first introduced in the mainland. Unfortunately, the model we used did not yield a pharmacoeconomic advantage due to the high cost. However, by February 2023, as IDegLira aimed to enter into Chinese health insurance negotiations, the price had been reduced from 499 CNY to 230 CNY. As a result, we re-conducted the CEA to reflect the lower price. Based on our perspective of medical insurance payments in China, the current price as of February 2023, which is 230 CNY, offers pharmacoeconomic benefits.

IDegLira was anticipated to reduce the incidence of diabetes-related complications and improve quality-adjusted life expectancy compared to liraglutide and degludec. A survival advantage was observed with IDegLira over Liraglutide (0.073 years). Lifetime costs were lower by CNY 27,945 on IDegLira than on Liraglutide therapy. A similar survival benefit was observed with IDegLira over degludec (0.068 years). Lifetime costs were lower by CNY 1196 on IDegLira than on degludec therapy. Therefore, IDegLira was deemed cost-effective compared to liraglutide and degludec with incremental cost-effectiveness ratios of Dominant per QALY gained, respectively, under the threshold of three times the GDP per capita in China. Cost-effectiveness evaluations based on clinical trial data on IDegLira indicate that it is likely to improve clinical outcomes and be cost-effective compared to several comparator regimens (basal insulin, basal-bolus insulin, and GLP-1 receptor agonists in combination with insulin) in patients with type 2 diabetes in the United States, the Netherlands, the Czech Republic, Sweden, the United Kingdom, and Spain [23, 34–38]. The pharmacoeconomic advantage should be credited to the enhancement in HbA1c levels achieved with IDegLira versus liraglutide, along with the negligible weight gain associated with IDegLira as compared to degludec [16, 17].

Our study has some limitations. Firstly, there have been no RCT studies of IDegLira combined with insulin glargine or multiple daily insulin injections (MDI) conducted in Chinese populations. Additionally, the pharmacoeconomics of IDegLira versus insulin glargine and MDI were not evaluated in this study. We hope that further RCTs conducted in Chinese populations can complement our results. Secondly, the data used in our study was obtained from the DUALI and DUAL II RCTs conducted in China. While real-world data may be more convincing, IDegLira has not yet been widely used in clinical settings. Therefore, we look forward to more real-world data on IDegLira becoming available. Lastly, we assumed that patients who received IDegLira, liraglutide, or degludec treatment would remain on that treatment until their HbA1c levels exceeded 7.5%, at which point they would be switched to basal-bolus therapy (IGlar OD + 3*IAsp). This assumption recognizes that intensification to basal-bolus therapy is necessary for patients to maintain glycaemic control over the long term [23]. There is literature available regarding the switch in protocols to IDegLira after five years of usage [37]. Nevertheless, we hold the belief that utilizing HbA1c as a conversion protocol marker presents greater suitability for clinical treatment.

In conclusion, IDegLira is a cost-effective hypoglycemic strategy that provides favorable clinical outcomes and cost reduction options for Chinese patients with type 2 diabetes. This is due to the improved HbA1c levels with IDegLira compared to liraglutide and the minimal weight gain with IDegLira as compared to degludec. Furthermore, we anticipate additional real-world data on IDegLira that can further support our findings.

### Electronic supplementary material

Below is the link to the electronic supplementary material.

Supplementary Material 1

Supplementary Material 2

Supplementary Material 3

Supplementary Material 4

Supplementary Material 5

## Data Availability

The datasets during and/or analyzed during the current study are available from the corresponding author on reasonable request.
